# Supplementary material for: HIV-1 suppression and rare dolutegravir resistance in antiretroviral-experienced people with HIV in Liberia
Source: Commun Med (Lond). 2025 May 9;5:164. doi: 10.1038/s43856-025-00875-x (PMC12064710; doi:10.1038/s43856-025-00875-x)

HIV-1 suppression and rare dolutegravir resistance in antiretroviral-experienced people with HIV in Liberia

James Soka Moses[1,2]*, Alice K. Pau[3], Safia Kuriakose[4], Greg Grandits[5], Cavan Reilly[5], Brad T. Sherman[6], Weizhong Chang[6], Lisheng Dai[6], Muhammad A Khan[6], Helene Highbarger[6], Moses Mannah[1], Johnathan McCullough[7], Carla Chorley[8], Isaac Morlu[1], Joseph Dorbor[1], Ophelia Talweh Bongolee[1], Rebecca Slewion[1], Esther Akpa[8], Barthalomew Wilson[1], April L Poole[3], Stacy L Kopka[8], Tracey Miller[8], Cecelia J Nuta[9], Christina Lindan[2], David Glidden[2], Jeffrey N. Martin[2], Kumblytee L Johnson[1], Robin L Dewar[6], Ian Wachekwa[1,9], Stephen A Migueles[3,10]

Author information: [1] Partnership for Research on Vaccines & Infectious Diseases in Liberia (PREVAIL), Monrovia, Liberia
[2]University of California San Francisco, San Francisco, California, USA
[3]National Institute of Allergy and Infectious Diseases (NIAID), National Institutes of Health (NIH), Bethesda, Maryland, USA
[4] Clinical Research Directorate, Frederick National Laboratory for Cancer Research, Frederick, Maryland, USA
[5] Division of Biostatistics, School of Public health, University of Minnesota, Minneapolis, Minnesota, USA
[6] Frederick National Laboratory for Cancer Research, Frederick, Maryland, USA
[7] Advanced BioMedical Laboratories, LLC, Cinnaminson, New Jersey, USA
[8] Clinical Monitoring Research Program Directorate (CMRPD), Frederick National Laboratory for Cancer Research, Frederick, Maryland, USA
[9] John F. Kennedy Medical Center, Monrovia, Liberia
[10]Axle Informatics, North Bethesda, Maryland, USA

***Corresponding author:** J. Soka Moses, Partnership for Research on Vaccines and Infectious Diseases in Liberia (PREVAIL), First Floor East Wing, John F. Kennedy Memorial Hospital, John F. Kennedy Medical Center, 21 Street, Sinkor, Monrovia, Liberia
Mobile: +231-770-102-855, SMoses@prevailcr.org

SUPPLEMENTARY MATERIALS

Table of Content

Supplementary Table 1: Comparison of participants with pVL measured at enrollment to those without enrolment pVL......................3
Supplementary Table 2: Characteristics at baseline of PWH on a DTG-based ART regimen 4
Supplementary Table 3: Factors associated with virologic failure in PWH on DTG-based ART at enrolment in HONOR (N=786).......5
Supplementary Table 4: Characteristics of PWH with VL ≥1000, successfully sequenced for drug resistance (N=70) 6
Supplementary Table 5: Characteristics of PWH with pVL >1000 copies/mL successfully sequenced by resistance to NRTI backbone7
Supplementary Table 6: Baseline characteristics of two participants on DTG-based ART with high-level resistance to DTG 8
Supplementary Figure 1: Study flow diagram...9

Supplementary Table 1: Comparison of participants with pVL measured at enrollment to those without enrolment pVL

	HIV viral load availability			
	Available	Unavailable	Total[1]	p-value
Characteristic	N=1,201	N=75	N=1,276	
Age, years, median (IQR)	42 (35-50)	40 (32-49)	42 (34-50)	0.22
Age groups				0.69
<15	37 (90%)	4 (9.8%)	41	
15-24	54 (95%)	3 (5.3%)	57	
25-49	793 (94%)	49 (5.8%)	842	
≥50	317 (94%)	19 (5.7%)	336	
Sex assigned at birth				0.46
Male	335 (95%)	18 (5.1%)	353	
Female	866 (94%)	57 (6.2%)	923	
Level of education[2]				0.91
None	240 (95%)	13 (5.1%)	253	
Primary-high sch	723 (94%)	46 (6.0%)	769	
Vocational-university	220 (94%)	13 (5.6%)	233	
BMI, kg/m^2, median (IQR)[2]	24 (21-28)	23 (20-27)	24 (21-28)	0.13
Hemoglobin (g/dL)[2,3]				0.59
≥11	968 (94.1%)	61 (5.9%)	1,029	
<11	227 (95.0%)	12 (5.0%)	239	
D-dimer (mg/dL)[2]				0.19
Normal (≤0·5 µg/mL)	794 (95%)	41 (4.9%)	835	
Elevated (>0·5 µg/mL)	385 (93%)	28 (6.8%)	413	
Years since HIV diagnosis, median (IQR)[2]	6.6 (2.6-11)	6.3 (2.9-12)	6.6 (2.6-11)	0.50
Years since starting ART, median (IQR)	6.1 (2.1-11)	6.2 (2.9-11)	6.1 (2.1-11)	0.56
pVL $\log_{10}$ (copies/mL), median (IQR)	1.3 (1.3-2.1)		1.3 (1.3-2.1)	
CD4 (cells/µL), median (IQR)	558 (362-779)	563 (285-857)	557 (360-782)	0.78
CD4 count (cells/µL)[2]				0.47
≥500	650 (95%)	35 (5.1%)	685	
200-499	375 (94%)	26 (6.5%)	401	
<200	93 (93%)	7 (7.0%)	100	
ART regimen at enrollment[4]				0.090
Efavirenz (EFV)/Nevirapine (NVP)	281 (97%)	10 (3.4%)	291	
Dolutegravir (DTG)-based regimen	894 (94%)	63 (6.6%)	957	0.56
Switched from NNRTI- to DTG-based regimen[5]	432 (94%)	28 (6.1%)	460	
Started ART on DTG-ART	449 (93%)	34 (7.0%)	483	
Lopinavir/ritonavir (LPV/r)/Nelfinavir (NFV)[6]	26 (93%)	2 (7.1%)	28	
Past ARV history				
Past treatment with EFV/NVP-based regimen	599 (94%)	39 (6.1%)	638	
Past treatment with Zidovudine (AZT)-regimen	243 (95%)	13 (5.1%)	256	
Past treatment with DTG-based regimen	1 (100%)	0 (0.0%)	1	
Past treatment with LPV /r-based regimen	23 (96%)	1 (4.2%)	24	

1: Values in this column are denominators for row statistics.
2: Missing data: Education (N=21 infants), BMI (N=4), Hemoglobin (N=8), D-dimer (N=22), Years since HIV diagnosis (N=12), CD4 count (N=90)
3: Normal haemoglobin (g/dL) for: female: ≥12 (pregnant: ≥11), male: ≥13, and children (0·5–4·9 years: ≥11, 5–11: ≥12, 12–14 years: ≥12).
4: Combined with 3TC+TDF (1167/1201 [97%]), 3TC+AZT (15/1201 [1.2%]), and 3TC+ABC (16/1201 [1.3%]).
5: The total row excludes 13 individuals (pVL<40=10, pVL 40-999=2, and pVL>=1000=1) with past exposure to LPV/r-based regimen.
6: Includes 3 participants on NFV-based ART.
Abbreviations: 3TC, lamivudine; ABC, abacavir; ART, antiretroviral therapy; ARV, antiretroviral; BMI, body mass index; IQR, interquartile range; ART, antiretroviral therapy; NNRTI, non-nucleoside reverse transcriptase inhibitor; pVL, plasma viral load; TDF, tenofovir.

Supplementary Table 2: Characteristics at baseline of PWH on a DTG-based ART regimen

	DTG-treatment experience		
	Started ART on DTG-based regimen	Switched from NNRTI- to DTG-based regimen	Total[1]
Characteristic	N=449[2]	N=432	N=881[2]
Age, years, median (IQR)	41 (32–49)	43 (37–52)	42 (35–50)
Age, groups			
<15	12 (60%)	8 (40%)	20 (2.3%)
15–24	35 (76%)	11 (24%)	46 (5.2%)
25–49	303 (52%)	280 (48%)	583 (66%)
≥50	99 (43%)	133 (57%)	232 (26%)
Sex assigned at birth			
Male	158 (64%)	88 (36%)	246 (28%)
Female	291 (46%)	344 (54%)	635 (72%)
BMI, kg/m^2, median (IQR)	24 (21–27)	25 (22–29)	24 (21–28)
Hemoglobin, g/dL			
<11	95 (55%)	79 (45%)	174 (20%)
≥11	352 (50%)	352 (50%)	70480%)
Missing	2 (67%)	1 (33%)	3 (0.3%)
D-dimer (µg/mL)			
Normal (≤0·5 µg/mL)	278 (49%)	291 (51%)	569 (65%)
Elevated (>0·5 µg/mL)	160 (54%)	137 (46%)	297 (34%)
Missing	11 (73%)	4 (27%)	15 (1.7%)
Years since HIV diagnosis, median (IQR)	2.8 (1.1–9.6)	9.1 (5.1–12)	6.3 (2.2–11)
Years since starting ART, median (IQR)	2.5 (0.6–9.4)	8.8 (4.9–12)	6.1 (1.9–11)
pVL, log_{10} copies/mL, median (IQR)	1.3 (1.3–1.9)	1.3 (1.3–2.1)	1.3 (1.3–2.0)
pVL groups, copies/mL			
<40	368 (50%)	361 (50%)	729 (83%)
40–999	57 (54%)	49 (46%)	106 (12%)
≥1000	24 (52%)	22 (48%)	46 (5.2%)
CD4 count, cells/µL, median (IQR)	533 (323–749)	587 (379–794)	560 (360–774)
CD4 count, cells/µL			
≥500	220 (48%)	241 (52%)	461 (52%)
200–499	146 (54%)	127 (46%)	273 (31%)
<200	40 (58%)	29 (42%)	69 (7.8%)
Missing	43 (55%)	35 (45%)	78 (8.9%)
Past AZT treatment	4 (2.1%)	190 (98%)	194 (22%)
Past EFV/NVP treatment	0	549 (100%)	549 (62%)
Past LPV/r treatment[1]	0	9 (100%)	9 (1.0%)

1:Values in this column are denominators for row statistics.
2: Excludes 13 individuals with past exposure to LPV/r-based regimen.
Abbreviations: ART, antiretrovial therapy; AZT, zidovudine; BMI, body mass index; DTG, dolutegravir; EFV, efavirenz; IQR, interquartile range; LPV/r, lopinavir/ritonavir; NNRTI, non-nucleoside reverse transcriptase inhibitor; NVP, nevirapine; PWH, persons with HIV; pVL, plasma viral load.

Supplementary Table 3: Factors associated with virologic failure in PWH on DTG-based ART at enrolment in HONOR (N=786)[1]

	Total	Total VF		
	(N=786)	(N=47)	Prevalence ratio (95% CI)	
Characteristics	n	n (%)	Unadjusted	Adjusted[2]
Age, years				
≥50	211	5 (2.4)	Ref	-
25–49	514	34 (6.6)	2.8 (1.1–7.0)	1.9 (0.8–4.9)
15–24	37	6 (16)	6.8 (2.2–21)	5.0 (1.6–16)
<15	24	2 (8.3)	3.5 (0.7–17)	-
Sex assigned at birth				
Male	217	16 (7.4)	-	-
Female	569	31 (5.4)	0.7 (0.4–1.3)	0.7 (0.4–1.3)
Education[3]				
Vocational-University	147	4 (2.7)	-	-
Primary-high school	462	33 (7.1)	2.6 (0.9–7.3)	2.6 (0.8–8.2)
None	165	8 (4.8)	1.8 (0.5–5.8)	1.6 (0.4–6.4)
Hemoglobin (g/dL)[3]				
≥11	642	26 (4.0)	-	-
<11	141	21 (15)	3.4 (2.3–5.0)	3.2 (1.6–6.2)
D-dimer (mg/dL)[3]				
Normal (≤0·5 µg/mL)	523	24 (4.6)	-	-
Elevated (>0·5 µg/mL)	251	21 (8.4)	1.8 (1.0–3.2)	1.0 (0.6–2.0)
Time since starting ART, years				
≤5	372	25 (6.7)	-	-
>5	414	22 (5.3)	0.8 (0.5–1.4)	1.3 (0.7–2.4)
CD4 count[3]				
≥500	435	13 (3.0)	-	-
200-499	226	19 (8.4)	2.8 (1.4–5.6)	2.5 (1.2–5.3)
<200	53	9 (17)	5.7 (2.6–13)	4.3 (1.8–11)
DTG-based ART regimen[4]				
Switched from NNRTI- to DTG-based regimen	383	22 (5.7)	-	-
Started ART on DTG-based regimen	392	24 (6.1)	1.1 (0.6–1.9)	0.7 (0.4–1.3)

1: We compared individuals with pVL<40 copies/mL (N=739) to individuals with pVL≥1000 copies/mL (N=47).
2: Adjusted for age, sex, education, hemoglobin, D-dimer, time on ART, and CD4 count.
3: Missing data: Education (N=12 infants), Hemoglobin (N=3), D-dimer (N=12), and CD4 count (N=72).
4: Excludes 11 individuals with past (pVL <40=10 and pVL ≥1000=1) with past exposure to LPV/r-based regimen.
Abbreviations: ART, antiretroviral therapy; CI, confidence interval; InSTI, integrase strand transfer inhibitor; LPV/r, lopinavir/ritonavir; NNRTI, non-nucleoside reverse transcriptase inhibitor; pVL, plasma viral load; PWH, persons with HIV; VF, virologic failure.

Supplementary Table 4: Characteristics of PWH with VL ≥1000, successfully sequenced for drug resistance (N=70)

Characteristic	N=70	%
Female sex	51	73%
Age, years, median (IQR)	37.0	30–43
Age, years		
<15	4	5.7%
15–24	8	11%
25–49	51	73%
≥50	7	10%
Years on ART, year, median (IQR)	6.1	2.0–9.9
ART regimen at enrolment		
EFV/NVP-based regimen	34	49%
DTG-based regimen	32	46%
Switched from NNRTI- to DTG-based ART	17	24%
Started ART on a DTG-based regimen	15	21%
LPV/r-based regimen	4	5.7%
HIV-1 clade		
CRF02_AG	56	80%
G	5	7.1%
C	3	4.3%
CRF06_cpx	3	4.3%
A	2	2.9%
F	1	1.4%
pVL $\log_{10}$ (copies/mL), median (IQR)	4.3	3.5–5.0
CD4 (cells/µL), median (IQR)	301	174–443
CD4 count (cells/µL)		
≥500	13	19%
200–499	33	47%
<200	20	29%
Missing	4	6%
Haemoglobin (g/dL)		
≥11	37	53%
<11	33	47%

Abbreviations: ART, antiretrovial therapy; DTG, dolutegravir; EFV, efavirenz; IQR, interquartile range; LPV/r, lopinavir/ritonavir; NNRTI, non-nucleoside reverse transcriptase inhibitor; NVP, nevirapine; PWH, persons with HIV; pVL, plasma viral load; VL, viral load.

Supplementary Table 5: Characteristics of PWH with pVL >1000 copies/mL successfully sequenced by resistance to NRTI backbone

Characteristic	No resistance to NRTI backbone N=27	Resistance to 1/both NRTIs N=43	All[1] N=70
Age, years, median (IQR)	38 (26–45)	37 (30–40)	37 (30–43)
ART regimen at enrolment			
DTG-based regimen[2]	19 (59%)	13 (41%)	32
EFV/NVP-based regimen[2]	7 (21%)	27 (79%)	34
LPVr/NFVr-based regimen	1 (25%)	3 (75%)	4
Years since HIV diagnosis, median (IQR)	2.1 (1.1–8.9)	8.2 (5.0–12)	7.3 (2.0–12)
Years on ART, median (IQR)	2.1 (0.6–8.9)	7.9 (4.0–11)	6.1 (2.0–9.9)
pVL $\log_{10,}$ copies/mL, median (IQR)	4.5 (3.5–4.9)	4.2 (3.4–5.1)	4.3 (3.5–5.0)
CD4 (cells/µL), median (IQR)	299 (193–414)	303 (156–464)	301 (174–443)
CD4 count (cells/µL)			
≥ 500	4 (31%)	9 (69%)	13
200–499	13 (39%)	20 (61%)	33
< 200	6 (30%)	14 (70%)	20
Missing	4 (100%)	0	4

1: Values in this column are denominators for row statistics.
2: Risk of HIVDR to the primary NRTI backbone, adjusted for time on ART, was lower in those on InSTI+2NRTIs compared to NNRTI+2NRTIs (unadjusted PR=0.5 [95%CI: 0.3–0.8]; adjusted PR=0·5 [95%CI: 0.3–0.8]).
Abbreviations: ART, antiretroviral therapy; DTG, dolutegravir; EFV, efavirenz; HIVDR, HIV drug resistance; InSTI, integrase strand transfer inhibitor; IQR, interquartile range; LPVr, lopinavir/ritonavir; NFVr, nelfinavir/ritonavir; NNRTI, non-nucleoside reverse transcriptase inhibitor; NRTI, nucleoside reverse transcriptase inhibitor; NVP, nevirapine; PR, prevalence ratio; PWH, persons with HIV; pVL, plasma viral load.

Supplementary Table 6: Baseline characteristics of two participants on DTG-based ART with high-level resistance to DTG

Characteristic	Participant 1	Participant 2
Age (years)	45-49	30-34
Sex	Female	Male
Hemoglobin (mg/dL)	≥11	≥11
Years since HIV diagnosis	14	0.7
Years on ART	14	0.1
Started ART on DTG-based regimen	No	Yes
pVL log_{10} (copies/mL)	3.9	4.1
CD4 count (cells/µL)	361	15
InSTI DRMs		
H51Y	-	+
T66A	+	-
G118R	+	+
E138K	+	-
R263K	+	-
NRTI DRMs		
M41L	+	-
K65R	-	+
D67G	+	+
K70R	+	-
M184I/V	+	+
T215F	+	-
NNRTI DRMs		
L100I	-	+
K101E/P	-	-
K103N	-	+
Y181C	+	-
Y188F/H/L	-	-
G190A	+	-
P225H	-	-
PI DRMs		
M46I	-	-
I54T/V	-	-
L76V	-	-

Abbreviations: ART, antiretroviral therapy; DRM, drug resistance mutation; DTG, dolutegravir; InSTI, integrase strand transfer inhibitor; NNRTI, non-nucleoside reverse transcriptase inhibitor; NRTI, nucleoside reverse transcriptase inhibitor; PI, protease inhibitor; pVL, plasma viral load.

Supplementary Figure 1: Study flow diagram

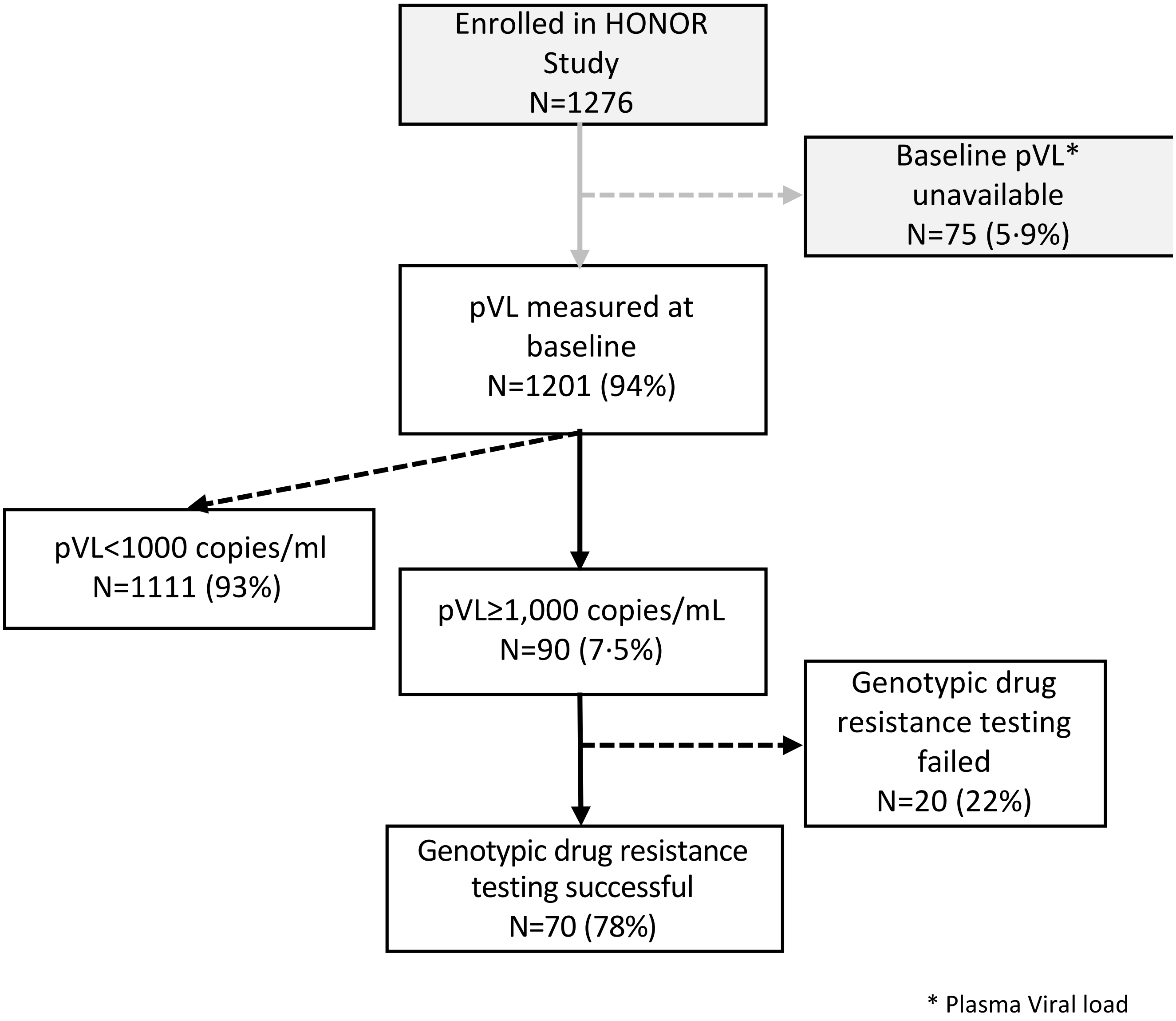

Supplement: Supplementary file 2 — Supplementary Materials [file 43856_2025_875_MOESM2_ESM.pdf]
