# Supplementary material for: HIV-1 suppression and rare dolutegravir resistance in antiretroviral-experienced people with HIV in Liberia
Source: Commun Med (Lond). 2025 May 9;5:164. doi: 10.1038/s43856-025-00875-x (PMC12064710; doi:10.1038/s43856-025-00875-x)
Supplement: Supplementary file 3 — Description of Additional Supplementary Files [file 43856_2025_875_MOESM3_ESM.pdf]

Description of Additional Supplementary Files

File name: Supplementary Data 1
Description: The source data for Figure 1
